# Evolution of moral expression in song lyrics

**DOI:** 10.1038/s41598-026-53778-9

**Published:** 2026-06-03

**Authors:** Vjosa Preniqi, Andreas Kaltenbrunner, Kyriaki Kalimeri, Charalampos Saitis

**Affiliations:** 1 Centre for Digital Music, Queen Mary University of London, London, E1 4NS UK; 2 Department of Engineering, Universitat Pompeu Fabra, Barcelona, 08018 Spain; 3 Universitat Oberta de Catalunya, Barcelona, 08018 Spain; 4 ISI Foundation, Turin, 10126 Italy

**Keywords:** Cultural and media studies, Cultural and media studies, Mathematics and computing, Psychology, Psychology

## Abstract

This study examines the evolution of moral expressions in popular music lyrics over six decades. Using the WASABI dataset (1960-2010) and Billboard year-end charting songs (1960-2023), we analyse temporal trends in moral narratives across artist genders and musical genres. Transformer-based language models fine-tuned for moral foundation prediction were applied to quantify ten moral dimensions derived from Moral Foundations Theory. Our findings reveal an increase in moral vices (e.g., Harm, Cheating, Subversion), and a decline in expressions of moral virtues (e.g., Care, Purity), alongside a rise in negative sentiment, anger, and disgust. We show that moral dimensions can be inferred by lyrical cues, such as thematic content, sentiment, and emotion, with predictive accuracy improving notably when models are trained on specific music genres. These findings reveal that shifts in lyrical morality co-occur with broader societal changes in values and identity, underscoring how popular music may serve as a cultural barometer for evolving moral norms.

## Introduction

Lyrics in music have been considered to serve several different functions^1^. They can influence listeners’ emotions and behaviours. When conveying positive messages, they can inspire and uplift, while negative or aggressive lyrics might have adverse effects on mood^2^. Historically, lyrics have also been used as a tool for social change. They have been employed as a means to stimulate social consciousness, motivate collective engagement, and mobilise listeners^3^. Additionally, music lyrics narratives have been used to promote peace^4^ and can serve as a potent and genuine voice for supporting women’s rights^5^. Often, the social, political, and cultural issues are reflected in the music lyrics of their time, which serve as a mirror to society, highlighting issues such as racial inequality, gender discrimination, and political unrest^6^.

In the landscape of popular music in the United States, there has been a notable increase since the 1980 s in lyrics that underscore self-focus and self-promotion, serving as a manifestation of the growing individualism prevalent in American society^7,8^. Blais-Rochette et al.^9^ examined bestselling singles across five decades in the U.S. and Canada, finding more cross-cultural similarities than differences in self-focus and group-focus words, with social connectedness words remaining consistently prominent. Over time, emotional words in lyrics have taken on a darker tone, while religious terms remained rare^9^. Studies tracking lyrical evolution show a consistent decline in positive sentiment (e.g., joy, serenity) and a rise in negative sentiment (e.g., anger, fear, sadness)^10–12^. As Brand et al.^12^ note, this shift may reflect both content bias, where charts favour darker lyrics, and cultural transmission biases, such as success or prestige bias, through which successful songs and artists are imitated.

Beyond these emotional and psychological dimensions, a substantial body of research has examined other aspects of lyrical and musical evolution. For example, the study by DeWall et al.^7^ on U.S. pop lyrics over three decades also revealed systematic shifts in word usage, such as an increase in first-person singular pronouns and a decline in words related to social connection, highlighting evolving linguistic structures alongside psychological change. Berger and Packard^13^ investigated lyrical novelty and typicality, demonstrating that songs which successfully balance familiarity with distinctiveness are more likely to gain popularity. Meanwhile, Mauch et al.^14^ used audio signal analysis to identify major stylistic shifts in U.S. pop music from 1960 to 2010, such as the emergence of hip-hop and electronic influences. While, Interiano et al.^15^ showed that popular songs have become increasingly homogeneous in terms of musical features like tempo, valence, and timbre.

In terms of the evolution of lyrical complexity, Varnum et al.^16^ found that the simplicity of lyrics in pop music increased over six decades (1958–2016). These analyses were further confirmed by a later study by Parada-Cabaleiro et al^17^ showing that songs have not only become simpler but also more repetitive. An alerting finding is the rise of gender bias and sexist content from 1960 to 2010, especially among male artists, for popular songs appearing in Billboard charts^18^. In fact, there is an ongoing debate about the ethical implications of certain types of lyrics, particularly those that may be seen as promoting violence, misogyny, or substance abuse^19^. A recent content analysis investigated the co-occurrence of violence, sexual content, and degrading terms toward women in music lyrics, highlighting the potential influence of such content on youth and young adults, and emphasising the need for media literacy education^20^.

Researchers have recently begun to explore psychological cues in music lyrics, with a growing focus on morality. Preniqi et al.^21^ showed that moral sentiment extracted from the lyrics to some extent can predict listeners’ moral values. These predictions are reinforced when moral features of lyrics are combined with audio and music features^22^. Further, Messick and Aranda^23^ found that moral values can explain a unique and significant portion of the variance in the lyrical preferences of different metal music sub-genre fans. For instance, a preference for lyrics celebrating metal culture and unity correlated with higher levels of loyalty foundation and increased extroversion.

In this study, we explore how moral expressions in song lyrics have evolved over time. We examine how moral expressions in song lyrics have changed by artist gender, then analyse their links with other lyrical attributes such as topics, sentiment, emotions, and lyrical similarity. Finally, we evaluate the predictability of moral dimensions from these cues, using both genre-specific and cross-genre models. We operationalise morality via the Moral Foundation Theory (MFT)^24^, which outlines five core moral traits, or foundations, divided into “virtue” and “vice” based on moral polarity. These foundations are *Care/Harm, Fairness/Cheating, Loyalty/Betrayal, Authority/Subversion,* and *Purity/Degradation*. The MoralBERT SL^25^ classifier was utilised to quantify these dimensions in lyrics. This model produces a probability score between 0 and 1 for each polarity, where higher values indicate greater confidence that the lyrical content expresses that moral dimension. MFT is a straightforward yet comprehensive model for understanding moral values, uniquely characterised by well-developed term dictionaries^26^. Moral foundations are intrinsically linked to emotions. Haidt and colleagues have shown that specific moral transgressions evoke distinct emotional responses^24^. For instance, violations of Care elicit compassion, Fairness violations provoke anger, Authority violations lead to resentment, Loyalty violations evoke rage, and Purity violations result in disgust^27^.

This study investigates two central research questions: (RQ1) How have moral values and narratives in song lyrics changed over time, and how do these trajectories differ by artist gender? (RQ2) To what extent are moral values associated with lyrical similarity, thematic content, sentiment, and emotions, and how well do these features predict moral dimensions both across genres and within specific genres?

To answer these questions, we examined lyrics from two large corpora: the broad WASABI dataset (1960–2010)^28^ and a targeted collection of Billboard Year-End charting songs (1960–2023). We note at the outset that both corpora carry important constraints. The WASABI dataset exhibits uneven temporal coverage, with a marked decline in available lyrics after 2010, and uneven genre representation, with several minority genres excluded from the analysis. The Billboard corpus, while extending temporal coverage to 2023, reflects only commercially successful chart entries and thus a narrow, popularity-filtered subset of music. These constraints are discussed in detail in the Methods section.

We first analysed the temporal evolution of moral foundations in lyrics. Then, we employed correlation analysis and machine learning methods to investigate the relationship between these moral foundations and other lyrical features, such as thematic content, sentiment, and emotion. By examining shifts in moral expressions and narrative themes across different periods, we gain valuable insights into how lyrics relate to evolving gender roles and broader cultural changes. This exploration underscores the cultural significance of song lyrics, highlighting their potential role in reflecting societal values and worldviews. Furthermore, since moral foundations play an important role in how people make decisions on various social issues^29,30^ and are closely linked to political leanings^31^, studying them in music lyrics can help address how music is used for persuasion in social and political campaigns. This includes raising awareness of pressing societal concerns, shaping public opinion, and mobilising collective action.

## Results

### Trends of moral expressions in lyrics: rising vices and declining virtues

**Fig. 1:**
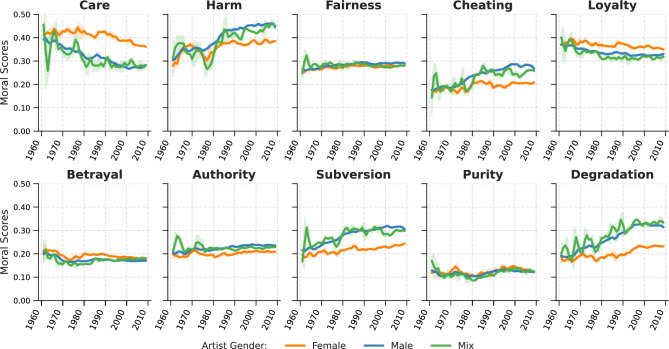
The overall lyrical trends of moral expressions by artist gender -Female (solo and groups), Male (solo and groups), Mix (groups that include both Male and Female members) - in WASABI lyrics. The plot lines, along with their 95% confidence intervals (shaded areas), were generated using a mean filter with a 2-year rolling window. The y-axis shows the mean MoralBERT SL probability score for each foundation, averaged across songs per year.

The following results are based on moral foundation scores predicted by the MoralBERT SL classifier^25^ for each song. Each score is a probability (0–1) indicating the confidence that a given moral dimension is expressed in the lyrics. Figure 1 reveals that the moral foundations of Care and Harm exhibit the strongest patterns of moral expression when looking at the average moral scores per year. Lyrics reflecting Care foundations appeared to be more present in female artists’ songs, while male artists’ song lyrics were more inclined towards the opposite polarity, Harm. Interestingly, over five decades, there has been a decline in songs expressing Care and a corresponding rise in songs expressing Harm, particularly after the 1970s.

It should be noted that the MoralBERT SL classifier performs unevenly across foundations; in particular, Fairness yields lower predictive accuracy^25^, and trends reported for this dimension should be interpreted with additional caution. This is consistent with our results, where Fairness scores appear nearly constant over time, showing only a modest increase. Whether this reflects a genuine absence of temporal change or a limitation of the classifier’s ability to capture this more nuanced foundation remains an open question. The opposite polarity, Cheating, showed an increasing trend, particularly among male and mixed-gender artists and groups.

Among other foundations, Loyalty showed a modest gender difference, with female artists expressing slightly higher scores throughout the period, alongside a gradual decline over time. Betrayal and Authority, by contrast, displayed relatively flat trajectories with limited gender differentiation; thus, it is difficult to determine whether this stability reflects genuine constancy in lyrical content or reduced classifier sensitivity. The clearest temporal patterns among the Binding-related dimensions were observed for Subversion and Degradation, both of which showed an increase over time, particularly among male and mixed-gender artists. Purity showed some fluctuation, rising during the 1970 s before gradually declining, with slightly higher expression among female artists.

To add statistical rigour to these visual observations, a series of Generalised Additive Models (GAMs) were fitted, with the results summarised in Table 1. The *Deviance Explained (%)* column indicates the percentage of variation in moral scores explained by year and artist gender, while *Magnitude (% change)* summarises the net change by contrasting fitted values at the start and end of the period. The *Trend Direction* column shows the general direction of observed change within the study period, determined by comparing model-fitted values at the start and end of the observation window. This does not imply the trend extends beyond the observed data. Statistical significance is assessed with two tests:   *(Year)* tests the significance of the main temporal trend, while   *(Gender *$\times$* Year)* tests the interaction effect, revealing whether the moral trend’s shape or slope evolved differently for male, female, and mixed-gender artists.

The GAM analysis supports the visual trends observed in Fig. 1, revealing that the temporal evolution of moral expressions in lyrics is statistically meaningful. Foundations associated with moral vices, Degradation, Harm, Cheating, and Subversion, showed the largest positive changes in magnitude (ranging from +40.91% to +52.25%) over the years and were statistically significant ($p<$ .001). Conversely, other moral foundations such as Care, Loyalty, Purity, and Betrayal exhibited significant gradual declines (from –11.26% to –24.38%, $p<$ .01 for Loyalty; < .001 for the other three). Fairness displayed a moderate but still significant increase (+9.6%, *p* < .01), while Authority showed a smaller (+5.46), marginally non-significant main temporal effect ($p=$ .105). However, Authority becomes more significant with the gender-year interaction ($p<$ .001). Indeed, all foundations showed significant interactions between year and artist gender (*p* < .001). Importantly, the models’ high *Deviance Explained* values (mostly exceeding 70% and over 85% for several foundations) indicate that these moral trajectories represent clear, structured temporal patterns rather than random fluctuations or noise.

**Table 1:** GAM results for Moral Foundations trends in WASABI lyrics. Overall trends were derived from predicted values between the start and end years. Significance: ** $p<0.01$, *** $p<0.001$.

**Moral Foundation**	**Deviance Explained (%)**	**Magnitude (% Change)**	**Trend Direction**	**Signif. (Year)**	**Signif. (Gender**$\times$**Year)**
Care	85.69	−24.38	Decline	***	***
Harm	88.32	49.13	Increase	***	***
Fairness	53.53	9.60	Increase	**	***
Cheating	82.73	47.99	Increase	***	***
Loyalty	83.40	−11.26	Decline	**	***
Betrayal	75.33	−11.43	Decline	***	***
Authority	71.76	5.46	Increase	.105	***
Subversion	88.25	40.91	Increase	***	***
Purity	69.56	−12.48	Decline	***	***
Degradation	91.73	52.25	Increase	***	***

**Fig. 2:**
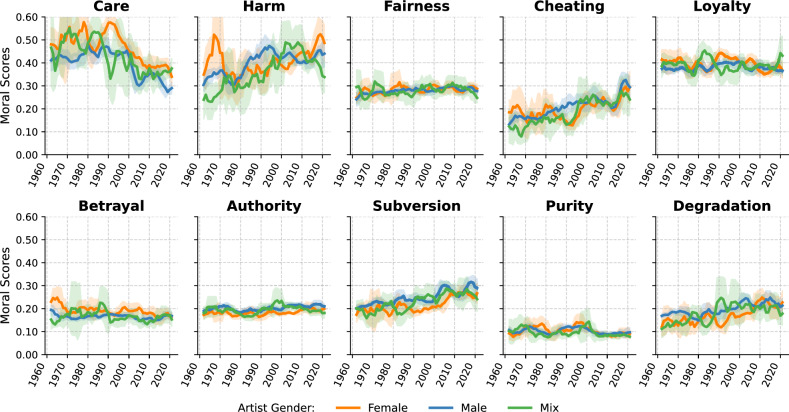
The overall lyrical trends of moral expressions in the Billboard end-of-the-year lyrics dataset. The plot lines, along with their 95% confidence intervals (shaded areas), were generated using a mean filter with a 5-year rolling window. *Note:* We applied stronger smoothing to the sparser Billboard dataset (window=5) compared to the denser WASABI dataset (window=2) to achieve comparable noise reduction. The y-axis shows the mean MoralBERT SL probability score for each foundation, averaged across songs per year.

Figure 2 shows the corresponding results for Year-End Billboard charting songs (1960–2023), which extends coverage beyond 2010. Comparing the large-scale trends from the WASABI corpus with the more focused Billboard dataset of popular songs reveals several common patterns. For instance, both datasets show a decline in the Care foundation over time, while Purity follows a similar trajectory, rising during the 1970 s, dropping in the 1980 s, increasing again in the 1990s–2000s, and then declining in the mid-2000s. Moreover, moral vices such as Degradation, Cheating, Subversion, and Harm appear to have increased across both datasets. In the Billboard data, these morally negative dimensions continue to rise steadily even after 2010, whereas Care and Purity show a continued downward trend. However, these cross-dataset comparisons should be interpreted with caution. Apparent late-period patterns in the WASABI data may partly reflect reduced data density rather than genuine cultural change. Similarly, trends in the Billboard data may reflect changes in commercial curation, radio programming, or streaming-era gatekeeping rather than shifts in the broader musical landscape.

Although gender differences are less pronounced than in the larger dataset, subtle distinctions remain. Song lyrics by female artists tend to express slightly higher Care scores overall, maintaining a small yet consistent lead over male and mixed-gender groups. In contrast, Harm narratives were more common in male-authored songs between the 1980 s and early 2000s. Interestingly, Harm scores among female artists rose during the late 2010s. While this temporal co-occurrence coincides with increased public discourse around social justice movements (e.g., MeToo, Black Lives Matter), our data do not permit causal attribution, and this pattern may equally reflect broader shifts in genre conventions or lyrical style during this period.

The GAM analysis of the Billboard dataset (Table 2) corroborates the broader moral shifts identified in WASABI. Although *Deviance Explained* values were found smaller (15–50%), this is expected given the reduced number of songs per year (87) and narrower topical diversity of charting music; the magnitudes of change remain substantial. Vice-related foundations such as Cheating, Degradation, Subversion, and Harm showed notable increases over time and were all statistically significant (ranging from +36.34% to +70.92%, $p<$ 0.05), while Betrayal expressions in lyrics showed a marginally non-significant decline (–22%, *p* = .783).   Virtue-related moral foundations displayed more mixed trends: Care and Purity showed significant gradual declines (–30.4% and –21.45%, respectively, *p* < .001), Authority and Fairness expressions increased moderately (+16%, *p* = .091; +7.4%, *p* = .102, respectively),  while Loyalty was found overall steady (+0.22%, *p* = .114). Importantly, all foundations exhibited statistically significant *Gender *$\times$* Year* interactions ($p<$ .01 for Purity; < .001 for all others), indicating differing moral trajectories across artist genders.

**Table 2:** GAM results for Moral Foundations trends in Billboard lyrics. Overall trends were derived from predicted values between the start and end years. Significance: ** $p<0.01$, *** $p<0.001$.

**Moral Foundation**	**Deviance Explained (%)**	**Magnitude (% Change)**	**Trend Direction**	**P-value (Year)**	**P-value (Gender**$\times$**Year)**
Care	42.89	−30.40	Decline	***	***
Harm	49.14	36.34	Increase	***	***
Fairness	18.05	7.40	Increase	.102	***
Cheating	46.38	70.92	Increase	***	***
Loyalty	15.22	0.22	Increase	.114	***
Betrayal	27.69	−22.08	Decline	.783	***
Authority	38.84	16.08	Increase	.093	***
Subversion	43.63	50.07	Increase	***	***
Purity	24.94	−21.45	Decline	***	**
Degradation	33.56	62.14	Increase	***	***

### How do moral foundation correlate with lyrical themes, affective cues and music genres?

**Fig. 3:**
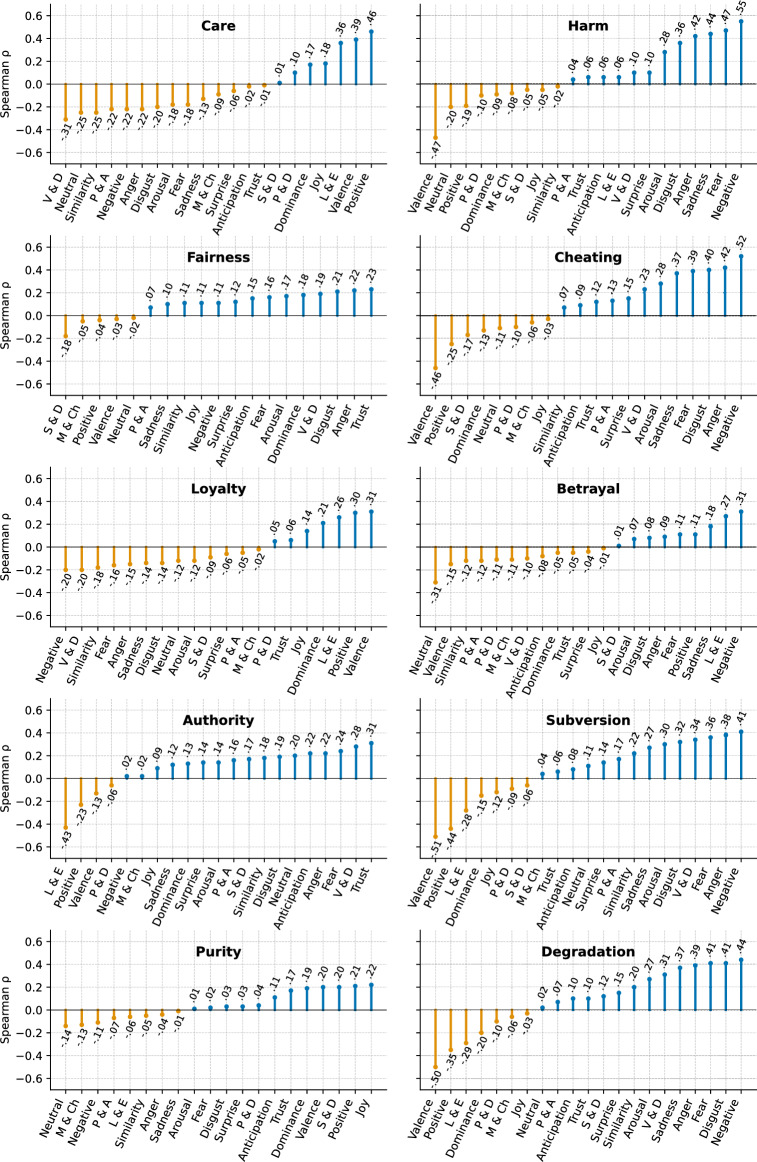
Spearman correlations between moral foundation scores and lyrical features. Statistically significant correlations are shown ($p<$ 2.38 $\times$ 10$^{-4}$). Abbreviations: L&E = Love & Emotions; V&D = Violence & Darkness; S&D = Spiritual & Dreamy; M&C = Movement & Change; P&D = Passion & Desire; P&A = Profanity & Aggression.

Here, we examine how moral cues in lyrics relate to other lyrical themes derived from Latent Dirichlet Allocation (LDA) topic modelling, a statistical method that identifies recurring word co-occurrence patterns in a text corpus to uncover latent thematic structures^32^. We also analyse affective and emotional signals in lyrics obtained via the lexical methods detailed in the Methods section. We restrict this analysis to the WASABI corpus, given its scale and suitability for correlation and regression, since no temporal component is required. We begin by presenting the correlation results. From the Spearman rank correlations depicted in Fig. 3, it can be observed that Care and Loyalty exhibited positive correlations with “Love and Emotions”, high lyrical valence, and positive sentiment, reinforcing their connection to empathy, connectedness, and social cohesion. Conversely, both foundations (Care and Loyalty) showed negative correlations with “Violence and Darkness”, negative sentiment, and emotions related to anger, sadness, and fear.

Interestingly, Fairness and Authority correlated positively with lyrics expressing trust but also with those conveying anger, disgust, and the “Violence and Darkness” theme. These moral dimensions reflected in lyrics might emphasise themes of justice, power struggles, and political criticism, either as calls for fairness or as critiques of authority. Purity, on the other hand, was correlated with positive sentiment and joy, as well as with the “Spiritual and Dreamy” topic, aligning with its conceptual ties to spirituality and divinity. In contrast, negative moral polarities such as Harm, Cheating, Subversion, and Degradation correlated positively with negative sentiment, lyrics expressing sadness, anger, and disgust, and the “Violence and Darkness” theme, while showing negative correlations with “Love and Emotions,” and positive valence.

Regarding lyrical similarity, songs expressing Care and Loyalty showed lower similarity in embeddings, suggesting that their lyrical expressions were more diverse. In contrast, Degradation and Subversion showed slightly higher similarity and more consistent linguistic patterns. It should be noted that although only statistically significant correlations ($p<$ 8.33 $\times$ 10$^{-4}$, Bonferroni-adjusted threshold as described in the Methods section) were reported in Fig. 3, some cases exhibit very small correlation coefficients. This can occur when analysing large datasets where even weak correlations may yield small *p*-values due to the large sample size.

Table 3 presents the prediction results of each moral foundation with XGBoost regression models, showing the proportion of variance ($R^2$) explained. The models used well-known lyrical cues as predictors, including LDA topics, emotions, sentiment, valence, arousal, dominance, and lyrical similarity. The models performed best for Care ($R^2 = .44$), Harm ($R^2 = .43$), and Subversion ($R^2 = .44$), indicating that these moral dimensions are predicted more reliably by lyrical characteristics. In contrast, Fairness ($R^2 = .15$) exhibited the weakest predictive performance. This is consistent with previous studies that predicted Fairness using user questionnaires^22,33^ and the MoralBERT approach for moral value prediction in lyrics ^25^, where this foundation consistently yielded the lowest prediction scores. This suggests that Fairness may be inherently more complex and nuanced, particularly in the context of music and lyrical content.

**Table 3:** Prediction results for each MFT, showing explained variance ($R^2 \pm$ standard deviation) using lyrical features as predictors. Top five predictors shown. *Abbreviations:* Feat. = Feature; L&E = Love & Emotions; V&D = Violence & Darkness; S&D = Spiritual & Dreamy; M&C = Movement & Change; P&D = Passion & Desire; P&A = Profanity & Aggression.

**MFT**	R$^2$ (Std.)	**Feat. 1**	**Feat. 2**	**Feat. 3**	**Feat. 4**	**Feat. 5**
Care	.44 (.0024)	L&E	Positive	V&D	P&A	P&D
Harm	.43 (.0021)	Negative	Fear	L&E	P&A	Valence
Fairness	.15 (.0035)	V&D	S&D	Trust	Dominance	L&E
Cheating	.34 (.0003)	Negative	V&D	Disgust	Anger	S&D
Loyalty	.24 (.0037)	L&E	Valence	Positive	V&D	S&D
Betrayal	.20 (.0030)	L&E	S&D	P&A	Negative	Neutral
Authority	.35 (.0020)	L&E	P&D	Trust	V&D	S&D
Subversion	.44 (.0051)	V&D	Valence	L&E	Anger	P&A
Purity	.30 (.0032)	S&D	Dominance	Trust	P&A	Positive
Degrad.	.43 (.0025)	V&D	Disgust	L&E	S&D	Valence

In Table 3, we have also presented the five most important features driving the predictions for each moral foundation. From this, it can be observed that lyrical topics such as “Love and Emotions”, “Spiritual and Dreamy”, “Violence and Darkness”, and “Profanity and Aggression” frequently emerged as top predictors, reinforcing the idea that thematic meanings are closely linked to moral expressions in music lyrics. Additionally, word-based sentiment, emotions, and affective features played a key role, with positive and negative sentiment, fear, disgust, and valence among the most influential predictors.

**Table 4:** Prediction results for each MFT, showing explained variance ($R^2 \pm$ standard deviation) using lyrical features as predictors, grouped by genre. Highlighted values mark the genre with the highest prediction accuracy for each foundation.

Genre-specific regression results (part I)			
**MFT**	**Country and Folk**	**Jazz and Blues**	**Metal**
Care	.38 (.005)	.42 (.002)	.30 (.021)
Harm	.38 (.007)	.38 (.008)	**.44 (.012)**
Fairness	.14 (.008)	.14 (.008)	**.15 (.018)**
Cheating	.27 (.008)	.30 (.012)	.24 (.007)
Loyalty	.22 (.006)	**.23 (.008)**	.18 (.013)
Betrayal	**.24 (.005)**	.21 (.016)	.11 (.006)
Authority	.37 (.010)	.31 (.009)	.31 (.010)
Subversion	.39 (.007)	.40 (.005)	.31 (.011)
Purity	.29 (.004)	.26 (.017)	.17 (.016)
Degradation	.36 (.004)	.35 (.010)	**.44 (.012)**

Genre-specific regression results (part II)			
**MFT**	**Pop**	**Punk**	**R&B and Soul/Funk**
Care	.40 (.006)	.32 (.007)	**.47 (.006)**
Harm	.37 (.004)	.37 (.008)	.39 (.006)
Fairness	.13 (.008)	.15 (.005)	.14 (.014)
Cheating	.28 (.004)	.27 (.014)	**.36 (.013)**
Loyalty	.21 (.006)	.13 (.007)	.22 (.010)
Betrayal	.18 (.010)	.12 (.013)	.20 (.012)
Authority	.29 (.007)	.23 (.016)	.35 (.013)
Subversion	.38 (.010)	.35 (.003)	**.46 (.020)**
Purity	.26 (.010)	.09 (.013)	.21 (.018)
Degradation	.36 (.007)	.34 (.013)	.35 (.006)

Genre-specific regression results (part III)			
**MFT**	**Rap/Hip Hop**	**Religious**	**Rock**
Care	.46 (.009)	.35 (.016)	.37 (.003)
Harm	.37 (.007)	.39 (.015)	.37 (.003)
Fairness	.12 (.006)	.11 (.014)	.14 (.005)
Cheating	.32 (.009)	.27 (.018)	.28 (.005)
Loyalty	.22 (.010)	.22 (.015)	.18 (.003)
Betrayal	.14 (.008)	.20 (.011)	.16 (.002)
Authority	.26 (.010)	**.48 (.016)**	.29 (.006)
Subversion	.40 (.006)	.38 (.013)	.37 (.003)
Purity	.25 (.027)	**.47 (.010)**	.22 (.006)
Degradation	.31 (.008)	.34 (.023)	.36 (.007)

Table 4 presents the cross-validated $R^2$ scores for each moral foundation across different music genres, demonstrating how genre influenced the predictability of moral expressions in lyrics. The results of this experiment showed that, in most cases, genre-specific models outperformed the models with mixed genres presented in Table 3.

For instance, models trained on R&B and Soul/Funk lyrics achieved the highest $R^2$ score for Care (.47). Even greater improvements were observed for Purity, which was better predicted in Religious music ($R^2 =.47$) compared to the overall dataset ($R^2 =.30$), suggesting a stronger moral association within faith-related lyrics. Similarly, Authority was most accurately predicted in Religious songs ($R^2 =.48$), reinforcing the idea that themes of hierarchy and obedience are more explicitly expressed in religious contexts. In contrast, Metal yielded the strongest predictions for Harm ($R^2 =.44$) and Degradation ($R^2 =.44$), likely reflecting the genre’s engagement with rebellious and darker narratives. These results highlight that, while some moral foundations were consistently predictable across genres, others were more dependent on musical style, with specific genres enhancing the models’ ability to capture particular moral expressions in lyrics.

## Discussion

Our temporal analysis of both the WASABI and Billboard datasets reveals a broadly consistent, decades-long evolution in lyrical morality. The findings indicate that expressions of moral vices such as Harm, Cheating, Subversion, and Degradation have generally increased, while moral virtues like Care and Purity have declined. This shift, which extends into the 2020s and varies across artist genders, highlights that vice-oriented and emotionally charged expressions have become increasingly prominent in mainstream lyrics. We emphasise that the analyses presented here are descriptive and correlational. Any references to historical events or social movements are intended strictly as contextual background regarding temporal alignment, rather than assertions of direct influence.

The findings also reveal observed associations between attributed gender categories and lyrical moral content, though these should be interpreted in light of the binary gender classification and the substantial gender imbalance in both datasets. Within these constraints, female artists were more frequently associated with virtues like Care and foundations reflecting relational conflicts, such as Loyalty and Betrayal. In contrast, male and mixed-gender groups more frequently featured negative polarities like Harm, Subversion, and Degradation. These gender-related patterns appear less pronounced in mainstream pop charts, suggesting a more homogeneous moral narrative in commercially successful music. These patterns are consistent with prior work showing that lyrics by male artists are more likely to contain profane, derogatory, and sexist content^18,34^, while female musicians more frequently reference themes of inspiration, cooperation, and diversity^35^.

Furthermore, the shift towards lyrical negative valence (moral vices) and emotional intensity may be associated with multiple factors. It could co-occur with content bias, where emotionally salient or transgressive themes are simply more successful and prevalent^12^. Alternatively, the trend may reflect the rise of countercultural movements. The emergence of genres like Hip-Hop, for instance, introduced a lyrical discourse that uses themes of systemic inequality and injustice, which our models identify as moral vices, to critique societal norms and challenge hegemonic structures^36^.

From a historical perspective, music has served as a tool for nonconformist movements and anti-war protests, reflecting societal values across different periods. Based on Stewart’s work^37^, the Vietnam War Era (1965–1975) and the “War on Terror” (2001–2010) represent two historical contexts where anti-war and pro-war songs had notable cultural presence. While a detailed investigation of moral expressions in music across historical periods falls outside our primary scope, our supplementary analysis shows that these expressions do vary across eras. Comparing yearly mean moral foundation scores across the Vietnam War Era, the Post-Vietnam War period (1976–2000), and the War on Terror, Kruskal-Wallis tests^38^ confirmed differences for most foundations. The largest changes between the Vietnam War Era and the War on Terror were observed for Degradation (+41.8%), Cheating (+36.8%), Subversion (+26.6%), and Harm (+25.1%), alongside a decline in Care (20.6%). Genre-specific comparisons indicate these shifts occurred individually within Rock, Pop, Country, and Punk, suggesting a broad cultural pattern rather than a compositional artefact (full results are available in Supplementary Tables S2–S5).

Our thematic and genre-specific analyses further demonstrated that moral expressions are closely embedded within broader lyrical and genre conventions, with genre-specific models consistently outperforming cross-genre ones. For example, lyrics expressing Harm and Degradation were best predicted within Metal music. As Hjelm et al.^39^ argue, Metal music thrives on being controversial, with its lyrical and visual elements designed to shock and challenge the status quo, often sparking “moral panics.” On the other hand, this type of music serves as a cultural touchstone for fans, creating a sense of belonging within a global, yet often marginalised, community. At the same time, it challenged mainstream values, offering an alternative worldview that embraced taboo subjects and questioned societal norms^39^.

Given that lyrical emotion recognition and sentiment analysis are already well-established in Music Information Retrieval (MIR) studies^40–42^, this work extended these approaches to the moral domain. The strong alignment we observed between moral polarity and emotional valence is consistent with Preniqi and colleagues’ work^22^, which similarly identified links between moral foundations and affective dimensions in music. As Rabinowitch^43^ highlights, music fosters social bonding and emotional synchronisation, making it a powerful force in shaping collective attitudes. By examining moral rhetoric in lyrics over decades, this work addresses a significant yet overlooked dimension of how popular music may both reflect and shape societal values.

### Limitations 

Some constraints of this work include its focus on Western, predominantly English-language popular music, which may limit the generalisability of the findings to other cultural or linguistic contexts with differing musical and moral frameworks. Additionally, the WASABI dataset exhibits an uneven temporal distribution, with a sharp decline in coverage after 2010. As a result, apparent trends in later years must be interpreted cautiously, as they may be artefacts of data scarcity rather than genuine shifts. The selective exclusion of genres with very low representation (<1.5%) may amplify the influence of dominant genres (Pop, Rock, Hip-Hop) in aggregate trends. The Billboard dataset, while extending temporal coverage, introduces its own bias by reflecting only chart-ranking songs shaped by editorial practices, commercial marketing, and streaming-era algorithms. Together, these characteristics mean that some of the reported temporal patterns, particularly those interpreted as cultural or societal shifts, may partly reflect data composition rather than underlying changes in musical culture.

Furthermore, our large-scale analysis relies on computational models to predict moral foundations. While these models have demonstrated high accuracy, they are not infallible, and their predictions inevitably introduce a degree of noise. The broad, consistent trends observed across decades suggest that these patterns are robust, but individual song-level predictions carry a margin of error. We also recognise that categorising complex lyrical narratives into discrete topics or moral foundations is an analytical simplification. Music is rich with nuance, irony, and metaphor that computational models may not fully capture. However, this approach provides a valuable macro-level view, revealing broad patterns that contribute an important “piece of the puzzle” for musicologists and cultural researchers. Finally, our correlational design cannot prove a cause-and-effect relationship. It remains unclear if music actively shapes societal morals or simply mirrors them, though the reality is likely a complex, bidirectional exchange.

## Methods

### Datasets - WASABI and billboard year-end collection

For this work, we utilise data from the Web Audio Semantic Aggregated in the Browser for Indexation (WASABI) dataset introduced by Meseguer et al.^28^. It comprises a knowledge base of 2 million songs, integrating metadata sourced from online music databases, analyses of song lyrics, and audio features. Additionally, the dataset supports the development of semantic applications designed to enhance the utility of this extensive resource (see study^28^ for full details).

WASABI was queried, following a similar approach to the work by Betti et al.^18^. The focus was on English song lyrics published between 1960 and 2010. The start year of 1960 was selected both because it marks the earliest period of substantial coverage in the WASABI dataset and because it aligns with the beginning of the Billboard Hot 100 era (established in 1958). Although WASABI includes data up to 2017, the analysis is limited to this period due to a significant decline in the number of available song lyrics after 2010. Although the dataset does not include the most recent years, we used it for its rich, well-organised metadata. With detailed information on artist gender, music genre, full lyrics, and release year, it offers a valuable foundation for analysing historical trends in song lyrics, especially in relation to moral expressions and narratives. To ensure sufficient representation, only artists with more than 10 published songs were included, resulting in a final dataset of 377,812 songs, including 7,131 solo artists and 4,294 group artists.

To complement the WASABI analysis and extend coverage beyond 2010, we gathered a corpus of songs from the Billboard Year-End Hot 100 charts spanning 1960–2023. This collection enables comparison between commercially successful charting repertoire and the broader catalogue in WASABI. Year-End chart entries were programmatically scraped from Wikipedia using Python’s BeautifulSoup library. Each entry’s title, credited artist(s), and chart year were extracted. The initial pass yielded 6,397 records; 159 rows with missing or malformed fields (owing to formatting variability, special characters, and markup inconsistencies) were manually audited against the original pages and corrected.

To match the artist information with WASABI, we extracted artist gender information through the MusicBrainz API (https://musicbrainz.org/), an open-source music metadata database. Table 5 shows the number of collected artists from WASABI and Billboard based on type and gender. Our gender analysis was limited to a binary classification (male/female) as the WASABI database does not include data for non-binary or other gender identities. This binary framing is a known limitation, as it does not capture the full spectrum of gender identities and may obscure important patterns among non-binary artists, particularly in more recent periods. Furthermore, the substantial imbalance in gender representation across both datasets (see Table 5) means that trends for female and mixed-gender artists are estimated from fewer observations and carry wider uncertainty. For methodological consistency, this binary approach was also applied to the Billboard dataset.

For lyric-based analyses, we retained only songs for which full lyrics could be obtained via the Genius API using title–artist queries. After excluding unavailable or ambiguous entries, the final subset comprised 5,580 songs (87 per year). Because Year-End rankings reflect commercial performance and editorial practices, this corpus is narrower and more volatile than WASABI, with fewer songs per year and a stronger popularity bias. This dataset is nonetheless well suited to our aims: it extends temporal coverage into the 2010s–2020s and offers a focused lens on how moral expressions evolve within mainstream popular music.

**Table 5:** Number of artists by gender and type in the WASABI and Billboard Year-End corpora (counts only). “Mix” denotes bands/groups including both male and female performers.

**Artist Type**	**Artist Gender**	**WASABI**	**Billboard**
Group	Female	306	137
	Male	3,281	973
	Mix	707	380
Solo	Female	2,361	317
	Male	4,770	712

### Lyrics moral expressions

The initial step of this work was predicting the moral foundation, including their polarities (e.g., vices and virtues), of each selected song from WASABI. For moral predictions, the MoralBERT SL presented by Preniqi et al.^25^ was utilised. MoralBERT SL consists of separate classifier models for each Moral Foundation, producing probability scores between 0 and 1. Values closer to 0 indicate lower confidence in the presence of a given moral value, while values nearer to 1 reflect higher confidence. The MoralBERT SL model was fine-tuned using both synthetic lyrics and out-of-domain social media data, achieving the highest overall performance in predicting moral values in lyrics compared to other out-of-domain classifiers (see^25^ for details). The 200-song lyrics dataset^25^ served as the ground truth, considering that these song lyrics were directly selected from the WASABI dataset. According to the MoralBERT SL classifier results^25^, the models for Care, Harm, Purity, Loyalty, and Subversion achieved the highest accuracy. Thus, it is expected that these foundations will also be the most reliable when predicting morality on a large-scale dataset. It should be noted that the predictions of moral values across lyrics in the WASABI dataset may introduce some noise into the moral trends due to the limitations of the MoralBERT SL models.

Temporal trends were computed by averaging moral scores across all songs per year, with gender-specific trends calculated by further grouping by artist gender. The analysis considers artists categorised as male, female, or mixed-gender. Within male and female categories, both solo artists and groups were included, while the mixed-gender category includes groups and collaborations that feature both male and female artists. A 2-year (for WASABI) and 5-year (for Billboard) rolling window approach, with a centred rolling mean and confidence intervals, is applied to highlight longer-term patterns and trends of moral expressions in lyrics.

#### Generalised Additive Models (GAMs) 

We selected GAMs due to their ability to model non-linear relationships without imposing a rigid parametric form^44^. For each of the ten moral foundations, a two-stage modelling approach was applied. First, to assess the main temporal effect, a GAM was fitted with the mean moral score as the target variable and a smooth term for the song’s release year as the predictor (*Score* *s*(*Year*)). The smoothing parameters were estimated automatically via the generalised cross-validation (GCV) criterion implemented in *pygam*, rather than set manually, to guard against overfitting. The $p-value$ for the smooth term indicates whether the observed change over time is statistically significant. Second, to investigate the moderating effect of artist gender, a separate interaction model was fitted, including a factor term for artist gender, a smooth term for year, and a tensor product interaction term $(Score ~ f(Gender) + s(Year) + te(Year, Gender))$. The interaction term tests the a priori hypothesis, motivated by prior literature on gender differences in lyrical content^18,34,35^, that moral trajectories may also differ across artist genders. This two-stage approach was specified before examining the results and applied identically to all ten moral foundations.

#### Music genres

In the WASABI dataset, music genres were extracted from DBpedia^28^ and provided as a list of genre tags. Often, songs were associated with multiple genre tags, some of which were ambiguous or redundant. To refine genre representation, only the first two genre tags for each song were considered in this study. These tags were then mapped to a custom genre dictionary designed based on the genre structure of Musicmap (https://musicmap.info/). The genre dictionary included genres such as Rock, Pop, Country, Hip-Hop, Folk, Metal, Soul/Funk, Blues, Jazz, Gospel, Punk, Electronic, R&B, Reggae, Soul/Funk, Techno, Trance, and World. Songs with genre tags from DBpedia that did not align with these categories were classified under “Other.” Additionally, genres with less than 1.5% representation, such as Reggae, Rock, Soul/Funk, Techno, Trance, and World, were excluded.

### Lyrics topics, affective traits, and similarity embeddings

To determine lyrics topics, the Latent Dirichlet Allocation (LDA)^32^ was utilised to uncover prevalent patterns within the topics of lyrics. LDA is one of the most commonly used methods for identifying latent thematic structures and is valued for its simplicity, high accuracy in topic modelling, and computational efficiency^45^. In this specific application, the input to the LDA model consists of a term frequency matrix derived from the song lyrics data corpus. The lyrics were preprocessed by converting text to lowercase, removing stopwords, and lemmatising tokens. To exclude overly general terms that might contribute to irrelevant topics, we excluded terms with a frequency exceeding 80%. To determine the optimal number of topics *k*, we employed the topic coherence metric ($C_v$ metric^46^) for models within the range $k \in [2,20]$ with a step size of 2. This range was selected following common practice in LDA-based analyses of song lyrics, where prior studies have typically identified between 3 and 10 broad thematic categories^21,22,47^. The coherence metric peaked at $k=6$, indicating that six topics best captured the thematic structure at a macro level. While a larger *k* could potentially yield more fine-grained topics, our goal was to identify broad, interpretable thematic categories that could be meaningfully related to moral dimensions across a large and diverse corpus. The topic labels were assigned based on the lyrics’ lemmas and extracted keywords; these include “Love and Emotions”, “Violence and Darkness”, “Spiritual and Dreamy”, “Movement and Change”, “Passion and Desire”, and “Profanity and Aggression”.

Furthermore, we analysed well-established lyrical characteristics such as sentiment and emotions from lexicon-based approaches. For determining whether lyrics are negative, positive or neutral, we used Aware Dictionary and sEntiment Reasoner (VADER), which was introduced by Hutto et al.^48^. VADER is a rule-based model tailored for social media content, and it provides intensity scores for sentiment. We then utilised the National Research Council Canada (NRC) Word–Emotion Lexicon^49^. The NRC lexicon contains 14,182 words, each assigned to specific emotions and sentiments. This lexicon is categorical, tagging each word with a single emotional state for emotion classification. To extract affective features such as valence, arousal, and dominance from the lyrics, we used the NRC VAD lexicon^50^, which provides human ratings for 20,000 words. These ratings were collected using the Best-Worst Scaling method, which has been shown to produce more reliable annotations than traditional rating scales by reducing bias and inconsistency among annotators^50^.

We quantified lyrical similarity using distilBERT embeddings^51^ and a Facebook AI Similarity Search (FAISS)^52^ index. For each song, we identified its ten nearest neighbours via approximate L2 distance search. Finally, we calculated the mean Euclidean distance between a song’s embedding and its neighbours (excluding self-comparisons) to derive an average similarity score, representing its lyrical mainstreamness within the dataset.

### Correlation and regression analysis

To further understand the connection between moral expressions and lyrical topics and affective features, we performed a Spearman rank correlation analysis. For this experiment, only correlation coefficients that met the Bonferroni correction^53^ were considered. This criterion represents a significance threshold of *p*-value $< .05 / (N_{\text {moral\_foundations}} \times N_{\text {lyrical\_features}})$, where $N_{\text {moral\_foundations}}$ represents the total number of moral foundations used as dependent features, and $N_{\text {lyrical\_features}}$ denotes the total number of lyrical features used as independent features (predictors).

Moreover, we conducted a regression analysis to assess how much variance in each moral foundation could be explained by widely studied lyrical cues, such as similarity, topics, word-based affect, emotions, and sentiment. Given the non-linear relationship between moral scores and these features, an XGBoost gradient-boosting regression model was employed. To ensure consistency in feature scaling, *MinMax()* normalisation was applied to features outside the [0,1] range.

Initially, an XGBoost (XGBRegressor) model was trained for each moral foundation using the full WASABI dataset. Then, song lyrics data were grouped by genre, creating smaller subsets for genre-specific regression models, in which lyrical topics, affective features, sentiment, emotion, and similarity scores served as predictors. The XGBoost models were configured with 500 trees, a maximum depth of 6, and a learning rate of 0.05, balancing predictive performance and the risk of overfitting. *L*1 ($\alpha = 0.5$) and *L*2 ($\lambda = 1.5$) regularisation were applied to control model complexity, and 80% feature subsampling per tree was used to enhance generalisability. For evaluation, 5-fold cross-validation was employed, computing $R^2$ scores for each fold and averaging the results.

## Supplementary Information

Supplementary Information.

## Data Availability

For the WASABI data access and API documentation, please refer to: https://wasabi.i3s.unice.fr/apidoc/. The Billboard dataset gathered and tailored for this study is available in the corresponding GitHub repository: https://github.com/vjosapreniqi/Lyrics-Moral-Evolution/tree/master/Dataset. Due to copyright re- strictions, full song lyrics retrieved via the Genius API cannot be redistributed. However, all derived data, including moral foundation scores, sentiment, emotion, and topic features, is provided within the repository. Detailed instructions and scripts for reproducing the full data collection process are also available.

## Code availability

All code used for data collection, feature extraction, and statistical analysis is openly available on GitHub at: https://github.com/vjosapreniqi/Lyrics-Moral-Evolution. The repository includes Jupyter notebooks for both the WASABI and Billboard datasets and scripts for reproducing the results reported in this manuscript.
