# Supplementary material for: Evolution of moral expression in song lyrics
Source: Sci Rep. 2026 Jun 3;16:19556. doi: 10.1038/s41598-026-53778-9 (PMC13294330; doi:10.1038/s41598-026-53778-9)
Supplement: Supplementary file 1 — Supplementary Information. [file 41598_2026_53778_MOESM1_ESM.pdf]

Supplementary Information

Lyrics Topics Extraction via LDA and Temporal Evolution

With respect to lyrical topics, the LDA topic modelling experiments indicated that the WASABI songs analysed in this study can be categorised into six universal themes ($k = 6$). Six lyrical topics were retrieved (as explained in the main body of the paper). The topic labels were assigned manually based on the lyrics' lemmas and extracted keywords. Table S1 presents the 15 most prevalent keywords for each topic.

In terms of lyrical thematic evolution, as depicted in Supplementary Fig. S1, songs centred on "Love and Emotions" have remained prominent, particularly among female artists. However, their prevalence began to decline in the mid-1990s

Table S1. The top 15 keywords generated by the LDA model for each topic. Offensive or profane words are censored with an asterisk. The names for these topics are proposed based on the keywords and lemmas of the songs clustered within each group. L&E = Love and Emotions; V&D = Violence and Darkness; S&D = Spiritual and Dreamy; M&C = Movement and Change; P&D = Passion and Desire; P&A = Profanity and Aggression.

L&E	V&D	S&D	M&C	P&D	P&A
love	kill	light	go	baby	sh*t
never	man	world	back	girl	f*ck
feel	dead	sky	come	wanna	b*tch
heart	black	soul	time	little	n*gga
say	cut	burn	home	want	rock
see	head	sun	day	good	a*s
make	war	eye	away	like	hit
want	eat	fire	take	boy	money
need	red	dream	long	make	wit
try	big	sing	run	right	hot
give	kid	rise	way	tonight	club
believe	f*cking	fall	say	shake	h*e
life	blood	shine	turn	need	beat
always	gun	rain	wait	crazy	game
think	white	star	night	bad	roll

and continued thereafter. In contrast, topics of "Violence and Darkness" have shown an upward trend, especially among mixed-gender groups and male artists. Similarly, "Profanity and Aggression" became increasingly prominent after the 1990s.

Songs with topics of "Movement and Change" were most prominent during the 1970s and 1980s, while the theme of "Spirituality and Dreamy" reached its peak in the mid-1960s to 1970s, particularly among group artists. This trend likely corresponds to the Hippie Era and the cultural influence of iconic bands such as The Beatles, Pink Floyd, and The Rolling Stones.

Established vs Rebellious Genres and Moral Polarities

This section presents additional experiments examining the average moral foundation scores across genres, conducted on both the WASABI and Billboard lyrics datasets. Supplementary Fig. S2 displays the average moral foundation scores across various music genres. Notably, Care and Loyalty scores are highest in genres such as Religious, R&B, Soul/Funk, Pop, Jazz, Blues, and Folk. Additionally, Purity is particularly significant in the Religious genre. In contrast, moral foundations with negative polarities, Harm, Cheating, Subversion, and Degradation, exhibit higher average scores in more "rebellious" or "non-conventional" genres, including Metal, Punk, Rap/Hip Hop, and, to a slightly lesser extent, Rock.

In the Billboard dataset, we extracted music genres via the MusicBrainz API. We then compiled and mapped the genres to match with Supplementary Fig. S3. Religious, R&B/Soul/Funk, and Jazz/Blues genres show the highest levels of Care, while Metal, Punk, and Hip-Hop/Rap dominate in Harm. Hip-Hop, Punk, and, to some extent, Metal also score higher in moral vices such as Cheating, Subversion, and Degradation. In contrast, Purity, Authority, Fairness, and Betrayal do not display any genre-specific dominance in Billboard data.

Moral Expressions Across Historical Periods

To descriptively assess whether moral expressions varied across historically distinct periods, we compared moral foundation scores across three temporal windows following Stewart's periodisation[37]: the Vietnam War Era (1965–1975), the Post-Vietnam War period (1976–2000), and the War on Terror (2001–2010). To avoid inflated statistical significance from the large sample size ($n > 370{,}000$ songs), we aggregated moral scores to yearly means and applied Kruskal-Wallis tests[38] on these year-level values (Tables S2 and S3). To identify when the steepest changes occurred, we additionally computed song-level means across a finer-grained four-period split, dividing the Post-Vietnam War period into two sub-periods: 1976–1989 and 1990–2000 (Table S4). Finally, to assess whether the observed shifts were consistent within individual genres or driven by changes in genre composition over time, we conducted genre-specific Kruskal-Wallis comparisons at the song level across the three main periods (Table S5). Rap/Hip Hop was excluded from this analysis as it had insufficient representation in the Vietnam War Era.

Across most genres, the direction of change was consistent: Care declined while Harm, Subversion, Degradation, and Cheating increased, reinforcing the pattern observed in the aggregate analysis. Metal showed the most extreme shifts, with

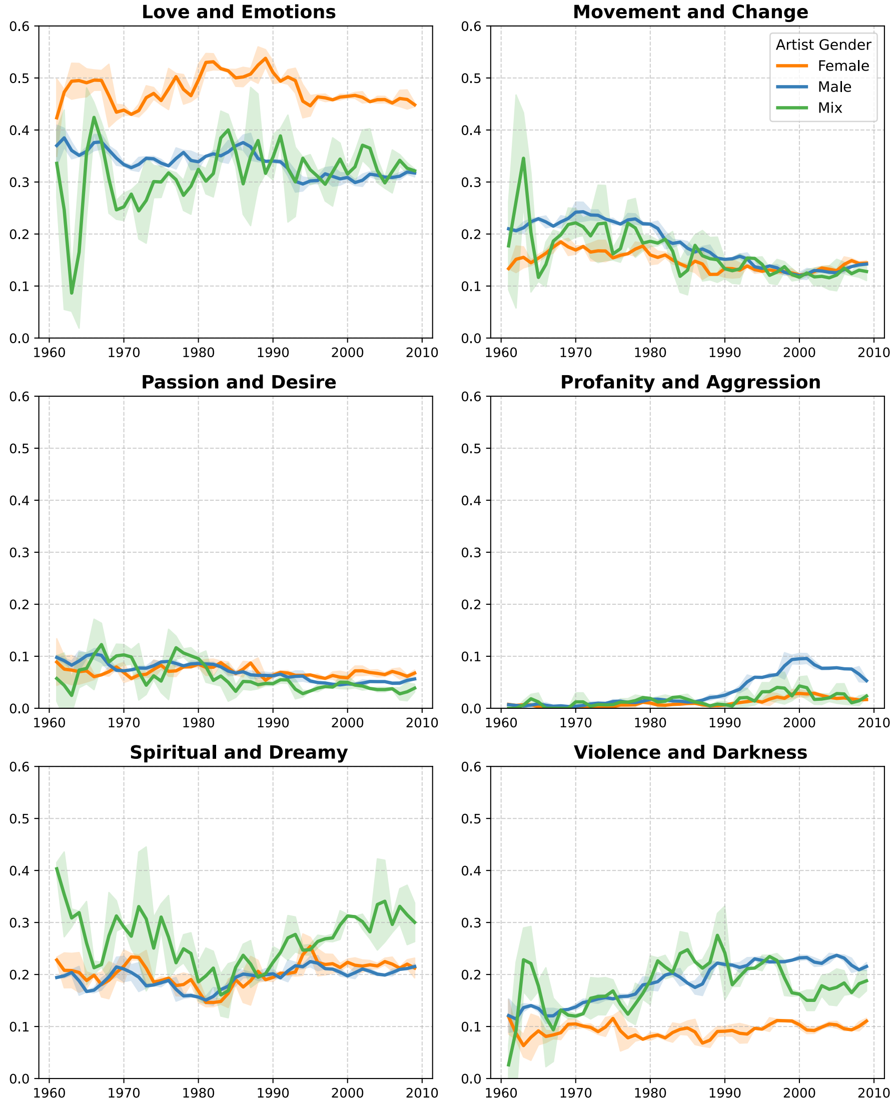

Figure S1. Trends of topics in lyrics. The plot lines, along with 95% confidence intervals, were generated using a mean filter with a 2-year rolling window.

Harm increasing by 63.1% and Degradation by 83.3% between the Vietnam War Era and the War on Terror. Jazz/Blues was a notable exception, showing no statistically significant changes for Care, Harm, or Subversion across periods

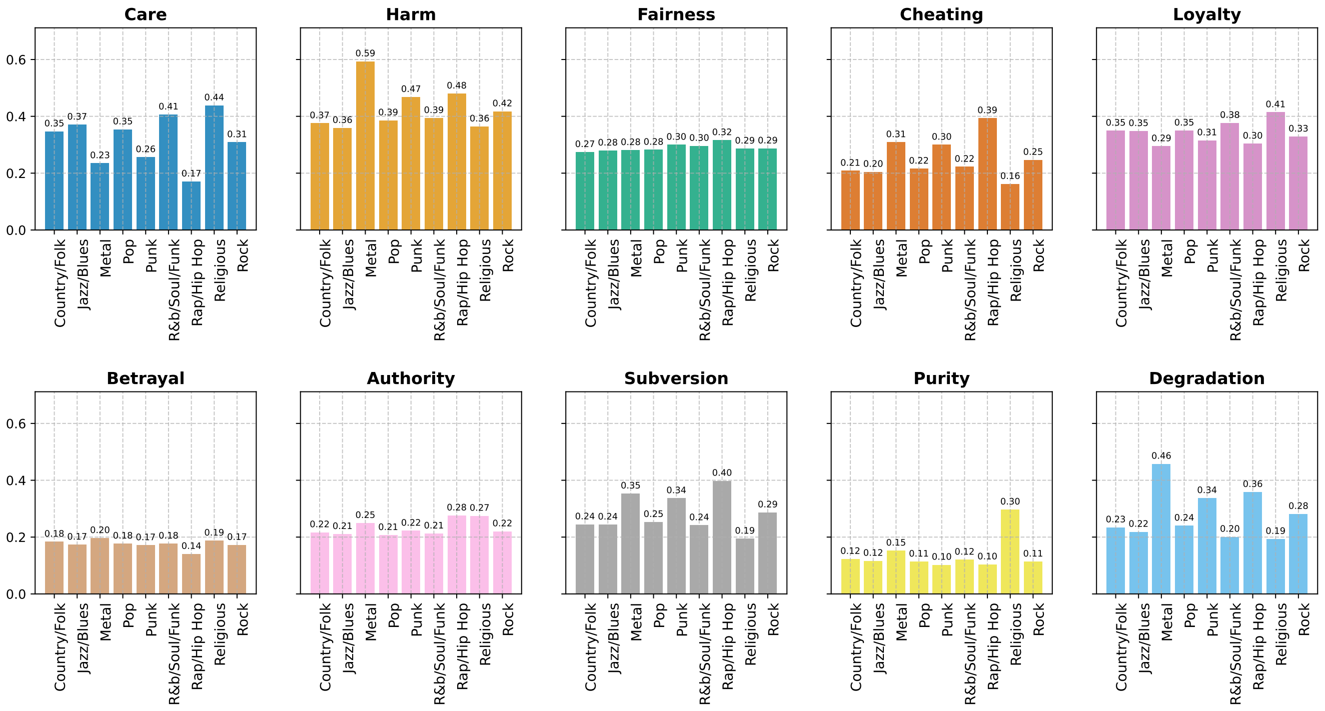

Figure S2. The average scores of moral expressions in WASABI lyrics across music genres.

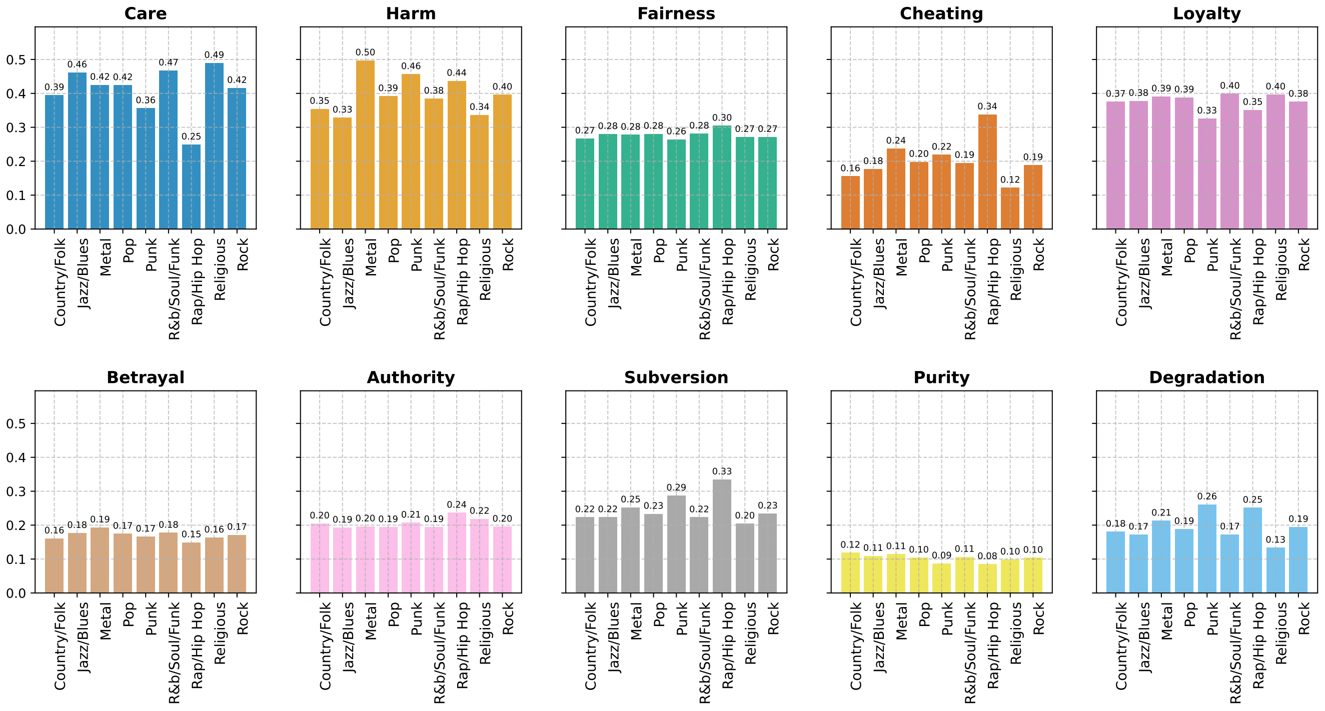

Figure S3. The average scores of moral expressions in Billboard lyrics across music genres.

Table S2. Yearly mean moral foundation scores (± SD) across historical periods in the WASABI dataset. VW = Vietnam War Era; Post-VW = Post-Vietnam War; WoT = War on Terror.

Foundation	VW Era (1965–1975)	Post-VW (1976–2000)	WoT (2001–2010)
	$n = 11$ years	$n = 25$ years	$n = 9$ years
Care	0.374 (±0.015)	0.325 (±0.018)	0.297 (±0.004)
Harm	0.350 (±0.007)	0.408 (±0.031)	0.438 (±0.006)
Fairness	0.273 (±0.006)	0.286 (±0.004)	0.288 (±0.001)
Cheating	0.190 (±0.006)	0.244 (±0.016)	0.260 (±0.005)
Loyalty	0.357 (±0.010)	0.337 (±0.005)	0.332 (±0.002)
Betrayal	0.178 (±0.013)	0.176 (±0.003)	0.173 (±0.001)
Authority	0.212 (±0.009)	0.222 (±0.008)	0.229 (±0.002)
Subversion	0.232 (±0.012)	0.281 (±0.013)	0.294 (±0.003)
Purity	0.114 (±0.007)	0.120 (±0.012)	0.126 (±0.003)
Degradation	0.212 (±0.016)	0.269 (±0.030)	0.301 (±0.004)

Table S3. Kruskal-Wallis tests on year-level moral foundation scores across three historical periods, with percentage change from the VW Era (1965–1975) to the WoT (2001–2010). Significance: * $p < 0.05$, ** $p < 0.01$, *** $p < 0.001$, ns = not significant.

Foundation	H-statistic	p-value	Sig.	VW Era	WoT	% Change
Care	32.29	< 0.001	***	0.374	0.297	−20.6%
Harm	27.82	< 0.001	***	0.350	0.438	+25.1%
Fairness	24.71	< 0.001	***	0.273	0.288	+5.4%
Cheating	29.25	< 0.001	***	0.190	0.260	+36.8%
Loyalty	25.93	< 0.001	***	0.357	0.332	−7.0%
Betrayal	4.61	0.100	ns	0.178	0.173	−3.1%
Authority	17.22	< 0.001	***	0.212	0.229	+8.4%
Subversion	31.13	< 0.001	***	0.232	0.294	+26.6%
Purity	5.71	0.057	ns	0.114	0.126	+10.5%
Degradation	24.91	< 0.001	***	0.212	0.301	+41.8%

Table S4. Song-level mean moral foundation scores across four historical sub-periods in the WASABI dataset. The Post-Vietnam War period (1976–2000) is split at 1990 to identify when the steepest shifts occurred.

Foundation	Vietnam War (1965–1975)	Post-Vietnam (1976–1989)	Post-Cold War (1990–2000)	War on Terror (2001–2010)
	$n = 37{,}896$	$n = 55{,}901$	$n = 108{,}530$	$n = 167{,}154$
Care	0.373	0.337	0.307	0.297
Harm	0.350	0.394	0.431	0.438
Fairness	0.274	0.286	0.286	0.288
Cheating	0.190	0.236	0.258	0.260
Loyalty	0.357	0.339	0.333	0.332
Betrayal	0.177	0.176	0.175	0.173
Authority	0.213	0.218	0.230	0.229
Subversion	0.233	0.276	0.290	0.294
Purity	0.115	0.111	0.131	0.126
Degradation	0.213	0.250	0.299	0.301

Table S5. Genre-specific moral foundation scores across historical periods for selected foundations (Care, Harm, Subversion, Degradation, Cheating). Percentage change (% Δ) is computed between the Vietnam War Era (VW) and the War on Terror (WoT). Sample sizes (n) denote the number of songs per genre in each period. Significance: * $p < 0.05$, ** $p < 0.01$, *** $p < 0.001$, ns = not significant.

Genre-specific period comparisons (part I)

Foundation	VW Era	Post-VW	WoT	% Δ	Sig.
**Rock** (n_{VW} = 13,718; $n_{Post\text{-}VW}$ = 61,054; n_{WoT} = 49,029)					
Care	0.342	0.307	0.297	−13.2%	***
Harm	0.352	0.415	0.440	+25.0%	***
Subversion	0.254	0.289	0.293	+15.4%	***
Degradation	0.231	0.282	0.296	+28.3%	***
Cheating	0.202	0.251	0.254	+26.0%	***
**Pop** (n_{VW} = 6,770; $n_{Post\text{-}VW}$ = 28,769; n_{WoT} = 33,080)					
Care	0.403	0.360	0.335	−17.0%	***
Harm	0.336	0.385	0.401	+19.2%	***
Subversion	0.216	0.252	0.263	+21.3%	***
Degradation	0.194	0.235	0.256	+32.3%	***
Cheating	0.170	0.219	0.224	+31.6%	***
**Metal** (n_{VW} = 378; $n_{Post\text{-}VW}$ = 12,070; n_{WoT} = 13,593)					
Care	0.310	0.238	0.229	−26.1%	***
Harm	0.378	0.573	0.617	+63.1%	***
Subversion	0.286	0.355	0.353	+23.4%	***
Degradation	0.254	0.455	0.466	+83.3%	***
Cheating	0.229	0.310	0.310	+35.3%	***
**Country/Folk** (n_{VW} = 15,487; $n_{Post\text{-}VW}$ = 28,802; n_{WoT} = 23,456)					
Care	0.380	0.343	0.325	−14.4%	***
Harm	0.357	0.376	0.388	+8.7%	***
Subversion	0.221	0.248	0.257	+16.2%	***
Degradation	0.205	0.233	0.259	+26.1%	***
Cheating	0.187	0.216	0.211	+12.8%	***
**Punk** (n_{VW} = 569; $n_{Post\text{-}VW}$ = 17,136; n_{WoT} = 12,225)					
Care	0.314	0.252	0.258	−17.8%	***
Harm	0.368	0.455	0.490	+33.2%	***
Subversion	0.289	0.335	0.340	+17.7%	***
Degradation	0.302	0.333	0.344	+13.8%	***
Cheating	0.223	0.297	0.307	+37.8%	***
**R&B/Soul/Funk** (n_{VW} = 2,692; $n_{Post\text{-}VW}$ = 10,353; n_{WoT} = 10,815)					
Care	0.443	0.417	0.385	−13.2%	***
Harm	0.365	0.392	0.403	+10.4%	***
Subversion	0.213	0.244	0.247	+16.0%	***
Degradation	0.172	0.199	0.210	+22.4%	***
Cheating	0.175	0.225	0.232	+32.8%	***
**Jazz/Blues** (n_{VW} = 7,105; $n_{Post\text{-}VW}$ = 16,783; n_{WoT} = 10,263)					
Care	0.375	0.368	0.366	−2.4%	ns
Harm	0.355	0.361	0.365	+2.9%	ns
Subversion	0.240	0.249	0.243	+1.2%	ns
Degradation	0.209	0.223	0.223	+6.8%	***
Cheating	0.194	0.209	0.207	+6.9%	***
